# A molecular view of the radioresistance of gliomas

**DOI:** 10.18632/oncotarget.21753

**Published:** 2017-10-11

**Authors:** Xuetao Han, Xiaoying Xue, Huandi Zhou, Ge Zhang

**Affiliations:** ^1^ Department of Radiotherapy, The Second Hospital of Hebei Medical University, Shijiazhuang, Hebei, China

**Keywords:** glioma, radioresistance, signaling pathway, microRNA, glioma stem cells

## Abstract

Gliomas originate from glial cells and are the most frequent primary brain tumors. High-grade gliomas occur ∼4 times more frequently than low-grade gliomas, are highly malignant, and have extremely poor prognosis. Radiotherapy, sometimes combined with chemotherapy, is considered the treatment of choice for gliomas and is used after resective surgery. Despite great technological improvements, the radiotherapeutic effect is generally limited, due to the marked radioresistance exhibited by gliomas cells, especially glioma stem cells (GSCs). The mechanisms underlying this phenomenon are multiple and remain to be fully elucidated. This review attempts to summarize current knowledge on the molecular basis of glioma radioresistance by focusing on signaling pathways, microRNAs, hypoxia, the brain microenvironment, and GSCs. A thorough understanding of the complex interactions between molecular, cellular, and environmental factors should provide new insight into the intrinsic radioresistance of gliomas, potentially enabling improvement, through novel concurrent therapies, of the clinical efficacy of radiotherapy.

## INTRODUCTION

Glial-derived tumors, or gliomas, are the most common primary tumors of the central nervous system. Approximately 82% of all gliomas are highly malignant WHO grade III and IV gliomas that have, even after standard treatment with surgery and radiochemotherapy, a 5-year survival rate of ∼10% [1]. Although surgery, radiotherapy and chemotherapy, i.e the mainstay of treatment for high-grade gliomas, have been refined by technological advances and results from clinical trials, glioma progression and recurrence is still the norm.

Radiotherapy is the most effective nonsurgical treatment for malignant gliomas, however, therapeutic efficacy is severely limited due to the high intrinsic radioresistance of glioma cells. Although multiple factors have been identified through intensive research, a detailed, integrative picture of the molecular network underlying glioma radioresistance has yet to be defined. It is critical to design novel strategies to sensitize tumor cells to radiotherapy and improve the prognosis and quality of life of glioma patients. Here we reviewed relevant articles from the last 10 years with the aim of summarizing the main molecular and cellular players determining the characteristic resistance of glioma cells to radiation, with a focus on signaling pathways, microRNAs, hypoxia, the brain microenvironment, and glioma stem cells (GSCs).

### Signaling pathways

#### AKT pathway

The AKT pathway, comprising the PI3K/AKT/mTOR nutrient sensing signaling cascade, is a critical regulator of cell growth, quiescence, proliferation, and longevity, and its dysregulation is a common tumorigenic event. Importantly, this pathway is required for promoting growth, proliferation and differentiation of adult stem cells, especially neural stem cells [2]. Overactivity of AKT signaling occurs in many cancers, especially in brain tumors [3], and is prominent in gliomas, where it has been associated with tumor progression, recurrence, radioresistance, and poor survival [4]. AKT hyperactivation may have several causes, including mutation or amplification of the epidermal growth factor receptor (EGFR) or the AKT gene, activation of upstream oncogenes, and PTEN repression [5]. Downstream components of the AKT signaling pathway includes NF-κB, GSK3, mTOR, MDM2, BAD, Caspase 9, among others; since many of these proteins are important regulators of survival and apoptosis, changes in their expression can trigger or augment radioresistance in tumor cells [6].

Multiple evidence links abnormal AKT signaling to radioresistance in glioma cells. It has been shown, for instance, that downregulated AKT signaling results in sustained, unrepairable DNA double-strand breaks (DSBs) following irradiation of U251 glioma cells [7]. Conversely, AKT activation could promote γH2AX foci resolution and enhance DNA damage repair (DDR) via homologous recombination (HR) and non-homologous end-joining (NHEJ) [8].These data demonstrate that AKT activation can modulate DDR in response to radiation, therefore affecting its efficacy.

Many proteins affect gliomas radioresistance through modulating of AKT signaling. Leucine-rich repeats and immunoglobulin-like domains protein 1 (LRIG1) is a transmembrane protein, widely expressed in human tissues and organs, that may function as a tumor suppressor [9]. It was reported that LRIG1 was downregulated in irradiated glioma U251R cells, and its overexpression significantly reduced EGFR signaling and AKT phosphorylation, increasing γH2AX foci formation and the rate of apoptosis [10].

EGFRvIII is the most common EGFR mutation, occurring concurrently with EGFR amplification in high grade gliomas [11]. Experiments in both U87 human glioma cells and primary mouse astrocytes showed that high level of EGFRvIII increased radiation resistance by promoting the rapid repair of radiation-induced DNA DSBs, an effect mediated by activation of the AKT pathway [11]. In addition, increased integrin-b1/EGFR heteroassociation was detected in glioblastoma compared with low grade gliomas. As this receptor complex induced activation of AKT signaling and increased glioma radioresistance, it was suggested that assessment of integrin-b1/EGFR association might be useful to predict radiotherapy outcome [12].

In conclusion, AKT pathway overactivation may promote glioma radioresistance by stimulating DDR and minimizing the deleterious effects of radiation-induced DNA DSBs. Although the specific mechanisms remain to be fully ecucidated, repressing AKT activation in glioma cells might be a powerful way to boost the efficacy of radiotherapy.

### Notch pathway

The Notch pathway mediates cellular communication between neighboring cells and plays a major role in regulating embryonic development during neurogenesis and neural differentiation [13, 14]. It consists of four receptors (Notch 1–4) and five ligands (Delta-like-1,-3,-4 and Jagged-1,-2), all of which are transmembrane proteins [15]. Upon ligand binding, Notch intracellular domain (NICD) is released into the nucleus by the γ-secretase complex, where it regulates the expression of multiple genes. The Notch cascade is an essential signaling pathway in GSCs [16]. After radiation exposure, Notch inhibition reduced cell growth, proliferation and clonogenicity, and promoted apoptosis, suggesting a close relationship between Notch signaling and radioresistance in glioma cells [17]. On the other hand, Wang et al. [18] found that blocking Notch signaling using γ-secretase inhibitors or Notch1/2-specific shRNA increased radiosensitivity, while activating it via NICD expression promoted radioresistance in GSCs. They further demonstrated that Notch inhibition enhanced radiosensitivity by reducing AKT activity and Mcl-1 levels, suggesting that the radioprotective function of Notch in gliomas is mediated by activation of the AKT pathway.

Additional evidence connecting the Notch pathway with glioma radioresistance comes from Shen et al. [19], who established a malignant host cell line (ihBTC2) induced by human SU3 glioma stem/progenitor cells that were orthotopically implantated into nude mice. They found that ihBTC2 cells were more radioresistant than SU3 cells, and that radiation exposure promoted Notch-1 and Hes1 mRNA expression, while increasing the levels of both phospho-AKT and the anti-apoptotic protein Bcl-2. In contrast, Notch pathway blockade using a γ-secretase inhibitor caused radiosensitization and promoted apoptosis in irradiated ihBTC2 cells.

These data suggest that Notch signaling is tightly related to glioma radioresistance, through a mechanism dependent on AKT activation. Although the exact molecular interactions remain to be clarified, the Notch pathway constitutes an attractive therapeutic target to increase glioma radiosensitivity.

### Wnt/β-catenin pathway

The Wnt/β-catenin signaling pathway is a crucial regulator of stem cell pluripotency and its dysregulation is often correlated with carcinogenesis and tumor invasiveness and radioresistance. In glioblastoma, the expression of β-catenin correlates with the degree of malignancy and may serve as a prognostic biomarker [20–22]. Using both immortalized glioblastoma cell lines and patient-derived, freshly dissociated glioblastoma cells, Kim Y et al. [23] developed an orthotopic mouse model system that mimics the radiation response of human glioblastoma. Drastic changes in Wnt pathway-related gene expression were found in xenograft tumors by transcriptomic analysis, represented by high levels of β-catenin and anti-apoptotic proteins in radioresistant tumors. On the other hand, Zhen et al. [24] also detected abnormal upregulation and nuclear accumulation of β-catenin in irradiated U87 glioma cells, and inhibiting the Wnt pathway reversed the invasive phenotype induced by radiation. In addition, several studies reported a relationship between the Wnt/β-catenin pathway and stem-like cell phenotype maintenance, differentiation inhibition and invasive potential, all of which are linked to radioresistance in glioma cells [25, 26].

This evidence suggests that β-catenin is a potential therapeutic target for overcoming resistance to glioma radiotherapy.

### ATM/Chk2/p53 pathway

The ATM/Chk2/p53 pathway is a key component of the cell cycle checkpoints and DDR machinery, as well as an important determinant in the molecular pathogenesis of gliomas [27]. It was reported that irradiation of glioma cells caused ATM kinase activation, phosphorylation of its downstream targets Chk2 and p53, and upregulation of DNA protein kinase (DNA-PKs), resulting in cell cycle arrest and apoptosis [28]. In this regard, Ross et al. [29] showed that enhanced ATM kinase activity led to more efficient DNA DSBs repair and selectively increased radioresistance in GSCs, rather than in their differentiated tumor cell counterparts. Conversely, inhibiting ATM potently radiosensitized GSCs. Meanwhile, Laura et al. [30] showed that the ATM kinase-specific inhibitor KU-60019 radiosensitized orthotopic glioma xenografts and increased survival. Notably, they showed that p53-mutant gliomas treated with KU-60019 are more radiosensitive than p53-wild type gliomas, suggesting that ATM kinase inhibition may be an efficient adjuvant therapy for glioma patients with mutated p53. These findings are further supported by two other studies that showed decreased radioresistance in glioma cells after inhibiting or silencing ATM kinase [31, 32].

Thus, signaling through the ATM/Chk2/p53 pathway promotes glioma radioresistance by activating DDR and inducing cell cycle arrest, suggesting that enhanced radiosensitivity and prolong patient survival might be achieved using inhibitors of ATM kinase or its substrates.

### STAT3 pathway

Signal transducer and activator of transcription 3 (STAT3) is at the center of a multifunctional intracellular signaling pathway involved in neural stem cell and astrocyte development [31]. The STAT3 pathway has a universal radioresistance-promoting function in many cancer types, including non-small cell lung cancer, squamous cell carcinoma, and head and neck carcinoma, as well as glioma [32]. In glioma tissues, phosphorylated-STAT3 (p-STAT3) levels correlate positively with tumor severity, suggesting a direct correlate between p-STAT3 status and glioma grade [32]. Accordingly, both *in vitro* and *in vivo* experiments showed that STAT3 inhibition promotes apoptosis and enhances radiosensitivity in glioma cells [33, 34].

In contrast, Chautard et al. suggested an inconspicuous relationship between STAT3 signaling and glioma radioresistance [7]. They tested STAT3 basal activation in the surviving fraction at 2 Gy (SF2) in 8 human malignant glioma cell lines, and found no correlation between STAT3 and SF2 in any cell line. Moreover, downregulation of STAT3 signaling using a specific inhibitor or a neutralizing gp130 antibody failed to confer radiosensitivity to glioma cells. This conflicting evidence calls for further investigation to better define the potential contribution of STAT3 signaling to radioresistance in glioma cells.

### Hedgehog pathway

The Hedgehog signaling pathway serves critical functions in cell differentiation during embryogenesis in the central nervous system, and in maintaining tissue homeostasis and repair after injury in adults [35]. In addition, it is remarkably active in CD133+ GSCs [36]; here, it promotes proliferation and prevents cell differentiation and apoptosis, therefore its inhibition enhances GSCs’ radiosensitivity [36, 37]. Specifically, it was shown that Hedgehog signaling activation in GSCs facilitated more efficient initiatiation of NHEJ-mediated repair of DNA DSBs, making cells more resistant to radiation exposure [36]. However, few studies have so far addressed the mechanisms by which Hedgehog signaling increases radioresistance in glioma cells.

**Figure 1:**
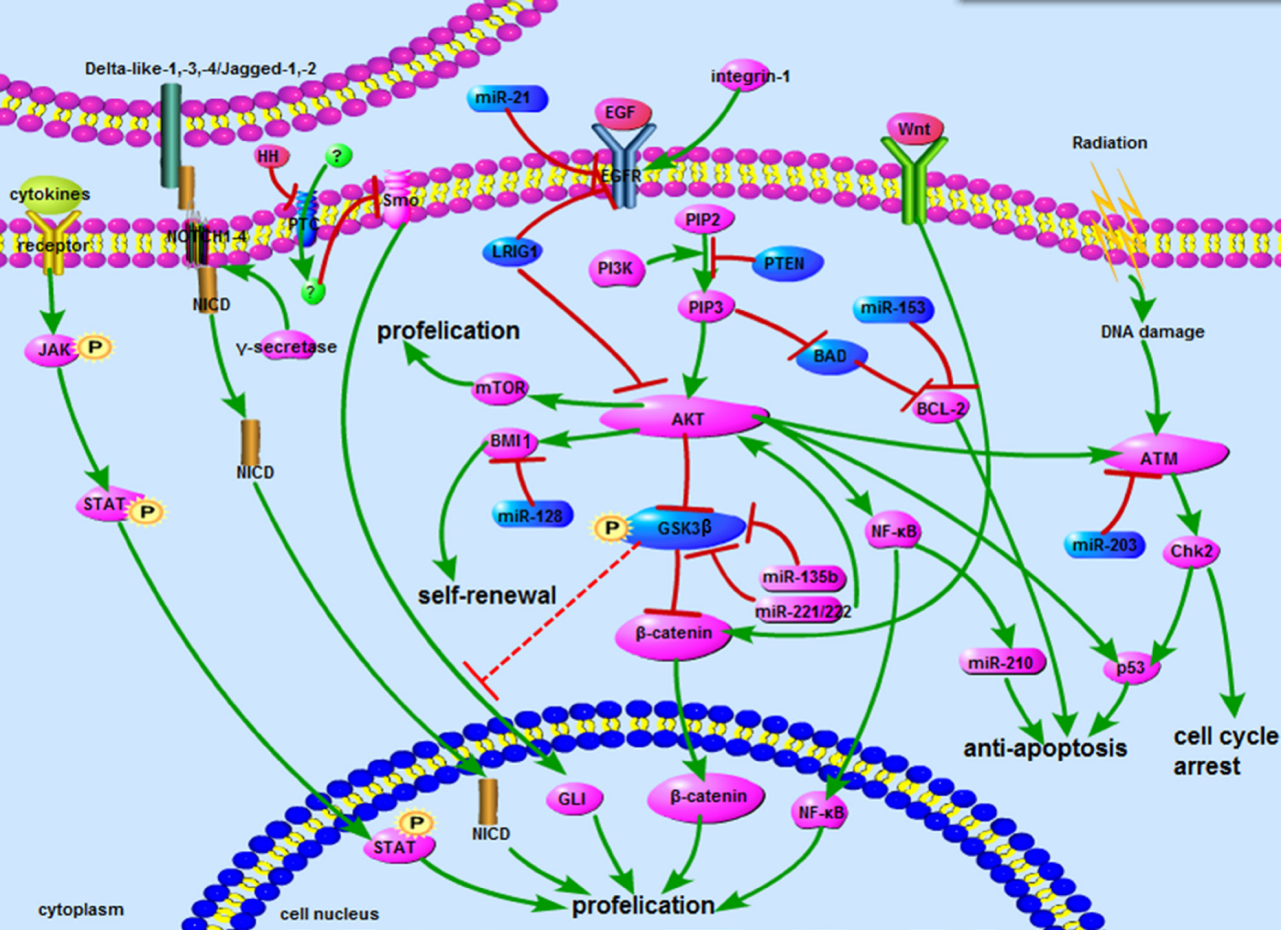
The network about signaling pathways The network includes AKT, Notch, Wnt, ATM, STAT3 and Hedgehog signaling pathways. Some microRNAs involved in signaling also showed above. Blue represents inhibitors of radioresistance, and purple represents activator. 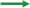: promote; 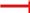: inhibit; 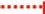: inhibit by surmise.

Although pathway-specific interactions underlie the contribution of particular signaling cascades to radioresistance in glioma cells, crucial crosstalk can take place between them via ubiquitous molecular players such as microRNAs. This complex, albeit still incomplete, signaling network is summarized in Figure 1. From Figure 1, we can find that the greater parts of reseaches related to glioma radioresistance were focus on AKT signaling. It seems a vital crossing that associated the whole network. There are tight links between these signaling pathways, especially in AKT, Wnt/β-catenin and ATM signaling. AKT activates the ATM signaling and increases the expression of β-catenin, which plays an important role in the formation of glioma radioresistance. Furthermore, many microRNAs regulate glioma radioresistance through acting with AKT signaling proteins, including EGFR, GSK3, Bmi. In addition, due to GSK3 is a recognized inhibitor of Hedgehog signaling pathway [38], we hypothesis that GSK3 might be a promising target to reduce the glioma radioresistance by downregulating Hedgehog signaling.

### MicroRNAs

MicroRNAs are non-coding, single-stranded RNA molecules, 21∼23 nucleotides in length, that are encoded by endogenous genes [39] and suppress mRNA translation [40]. Notably, some microRNAs have also been shown to modulate transcriptional gene activation or silencing, and evidence suggests that a single microRNA can modulate numerous target genes, while a single gene can be modulated by multiple microRNAs. Several microRNAs were identified as significant regulators of carcinogenesis, and may act as biomarkers or therapeutic targets in radiotherapy. Some prominent examples of microRNAs influencing glioma radioresistance are described below.

### MicroRNA-128

MicroRNA-128 (miR-128), a brain-specific miRNA, is significantly downregulated in glioblastoma [41]. Pierpaolo et al. [42] discovered that miR-128 could directly interact with mRNAs of Bmi-1 and SUZ12, two members of the Polycomb Repressor Complex (PRC), which has oncogenic activity in glioblastoma and is associated with cancer stem cell self-renewal and radiation resistance. Overexpression of miR-128 rendered GSCs more sensitive to radiation by preventing DDR, an effect alleviated by increasing expression of PRC genes. Another study showed that high doses of radiation reduced miR-128 levels and increased Bmi-1 expression in glioma U87 cells, which may promote escape from radiation-induced cellular senescence and confer instead radioresistance [43].

### MicroRNA-153

MicroRNA-153 (miR-153), which is also enriched in brain [44], has the ability to reduce stemness properties such as self-renewal and induce cell apoptosis by targeting Bcl-2 and Mcl-1. Abnormally downregulated miR-153 was detected in human glioblastoma specimens and in CD133+ cells isolated from corresponding tumorsphere cultures [46, 47]. Overexpression of miR-153 suppressed the synthesis of nuclear factor erythroid 2-related factor 2 (Nrf-2), a transcription factor upregulated in response to oxidative stress, leading to cell apoptosis and enhanced differentiation and radiosensitivity in GSCs [45]. Accordingly, the survival of mice bearing human glioma cells overexpressing miR-153 was significantly increased, while Nrf-2 overexpression made the mice more resistant to radiation [45].

### MicroRNA-210

High expression of microRNA-210 (miR-210) can be observed in some malignant tumors, especially in glioblastomas [44, 46]. MiR-210 is involved in cell survival, stemness maintenance, and hypoxia adaptation [47, 48], and its expression is modulated by hypoxia inducible factor (HIF) and nuclear factor κB (NF-κB) [49]. Yang et al. [50] found that knockdown of miR-210 radiosensitized hypoxic GSCs. In addition, knockdown of miR-210 increased the apoptotic rate and reduced the antioxidant capacity of gliomas. These data suggest that glioma progression might be halted by combining miR-210 inhibition and radiotherapy.

### MicroRNA-21

MicroRNA-21 (miR-21) levels were found to be significantly increased in both malignant glioma cell lines and tissues, and to promote cell survival, tumor growth, and chemo- and radioresistance [51]. Zhou et al. [52] showed that miR-21 is upregulated in radioresistant SHG-44(R) glioma cells relative to parental SHG-44 cells, and miR-21 knockdown combined with radiation enhanced apoptosis. Additional studies in glioma cell lines and xenograft tumor models revealed that miR-21 knockdown decreased the expression of EGFR, cyclin D, phospho-AKT and Bcl-2, increased autophagy and apoptosis, and induced cell cycle arrest [53, 54].

### MicroRNA-203

MicroRNA-203 (miR-203) is another cancer-associated microRNA dysregulated in many malignant tumors. In gliomas, its expression is significantly lower than in normal brain cells and correlates with WHO grade and prognosis [55]. Genetic analyses identified ATM was a direct target of miR-203, an interaction that results in inhibition of DDR by HR [56]. Chang’s research [57] also uncovered that miR-203 sensitizes glioma cells to radiation by modulating AKT and STAT3 pathways to repress DDR.

### MicroRNA-135b

MicroRNA-135b (miR-135b) expression was found to be increased in the radioresistant human glioblastoma cell line U87R compared to parent U87 cells [58]. Knockdown or overexpression of miR-135b increased or reduced, respectively, radiosensitivity by directly regulating GSK3β. To determine the relationship between miR-135b and GSK3β levels and radioresistance in glioma patients, expression data were obtained in glioblastomas and normal brain tissue. Results showed that miR-135b was significantly upregulated, while GSK3β was downregulated, in recurrent tumors compared to primary ones after treatment with ionizing radiation. These results indicate that miR-135b and GSK3β levels are tightly related to the radiotherapeutic effect. Therefore, miR-135b might be an important biomarker to assess radioresistance, as well as a promising therapeutic target in the treatment of malignant gliomas.

### MicroRNA-221/222

MicroRNA-221/222 (miR-221/222) is highly influential in the modulation of the DNA damage response. Li et al. [59] showed that radiation-induced c-jun gene activation promoted the transcription of miR-221/222 in glioblastoma cell lines, leading to decreased GSK3β expression, AKT activation, and increased DDR resulting in enhanced radioresistance. Furthermore, in a glioblastoma xenograft mouse model, both tumor volume and expression of the catalytic subunit of DNA-PK (DNA-PKcs) were significantly reduced after miR-221/222 knockdown.

### Glioma stem cells

GSCs are pluripotent cells with self-renewal capability. They are able to proliferate continuously and form neurospheres, and possess characteristic biomarkers such as CD133, nestin, and SOX2 [60, 61]. Studies have suggest that CD133+ GSCs might represent the radioresistant tumor cell population that survives radiotherapy [62], and a main cause of recurrence in primary glioblastomas [63]. CD133+ GSCs express high levels of autophagy-related proteins [64], and are thought to be a critical factor for gliomas treatment failure [65–67]. Numerous mechanisms seem to underlie the unique radioresistance exhibited by GSCs. While some of them where described above, there are several others acting to reduce or prevent apoptosis and enhance the cells’ DDR capacity [68, 69] by activating, for instance, DNA checkpoint proteins such as ATM, Chk1 and Chk2 [70].

Research has shown that some stemness markers such as L1CAM (CD171) [71], Bmi-1 [72], SOX2 [73], and CD44 [74] can also regulate glioma radioresistance. L1CAM (CD171), a cell surface molecule, is preferentially expressed on GSCs and contributes to tumor growth and survival [75]. Cheng et al. [71] found that L1CAM enhances GSCs radioresistance by increasing phosphorylation of ATM and Chk2. Meanwhile, L1CAM knockdown attenuated the G2 arrest induced by radiation, reduced DNA repair capacity, and accelerated cell death, while decreasing the formation efficiency and the size of GSC tumorspheres. These data suggest that L1CAM is a another potential molecular target to overcome radiation resistance in glioma radiotherapy.

Bmi1, another glioma stem cell marker, belongs to the Polycomb-group gene family, which can promote self-renewal and cellular proliferation [76]. Facchino et al. [77] found that Bmi1 is redistributed into chromatin after radiation, where it combines with DNA DSB response proteins. Knockdown of Bmi1 impairs this recruitment, induces cell apoptosis, and inhibits cell proliferation, thereby sensitizing GSCs to radiation [77]. As mentioned above, another mechanism by which Bmi-1 can mediate acquired radioresistance is after downregulation of its repressor, miR-128, concurrently with an increase in its own expression following radiation [42, 43]. Therefore, targeting Bmi-1 may provide therapeutic benefits to glioma patients undergoing radiotherapy.

SOX2 is a vital regulator of self-renewal capacity in both normal and tumor stem cells. Overexpression of SOX2 in glioblastoma cells induced GSC-like properties, suggesting a prominent role of SOX2 in glioma progression and recurrence [78]. Forkhead box M1 (FoxM1), an oncogenic transcription factor, promotes clonogenic growth, stem-like properties, and GSCs radioresistance by stimulating the expression of SOX2 [73]. Both FoxM1 and SOX2 are clearly increased in GSCs after irradiation. FoxM1 knockdown has been shown to decrease SOX2 expression, impair cell growth and clonogenicity, and sensitize GSCs to radiation-induced cell death.

CD44 expression in glioblastomas is correlated with cancer stem cell phenotype, tumor aggressiveness, and poor survival [74]. Based on gene expression signatures, gliomas are classified in two subtypes, termed proneural (PN) and mesenchymal (MES) [79, 80]. The PN subtype is enriched in CD133+ GSCs and shows significantly improved survival after radiotherapy treatment. In contrast, the MES subtype is enriched in CD44+ GSCs and shows limited benefit from radiotherapy [80]. It was found that NF-κB activation caused both MES differentiation and CD44+ expression in GSCs, suggesting that NF-κB-targeted therapies may be effective to increase radiosensitivity in this particular glioblastoma subtype [79].

In view of the multiple mechanisms underlying the intrinsic radioresistance exhibited by GSCs, novel therapies targeting these cells might provide a long-sought breakthrough for glioma radiotherapy.

### Hypoxia

Hypoxia is associated with tumor angiogenesis and invasiveness, therapeutic resistance, and poor prognosis [81]. A few studies have shown close-knit association between hypoxia and radioresistance in glioma [82–85]. Kessler et al. [84] discovered that DNA-PKcs, acting on the NHEJ pathway of DDR, regulates HIF-1α expression and confers radioresistance to gliomas. Accordingly, silencing DNA-PKcs expression in four human glioma cells lines reduced HIF-1α levels and increased their radiosensitivity. Two Researches found that hypoxia can increase cell proliferation, self-renewal, and promote stemness maintenance in GSCs with intrinsic radioresistance [82, 83]. In the hypoxic microenvironment, the expression of hypoxia inducible factors (HIF-1α and HIF-2α) is elevated, with HIF-1α expression detected both in GSCs and non-GSCs, and HIF-2α expression observed in GSCs exclusively. Notably, overexpression of HIF-2α in non-GSCs transformed them into GSCs, and HIF-2α, but not HIF-1α, expression correlated with survival in high-grade glioma patients [83]. Although the detailed molecular interactions remains unclear, It is not hard to find the pivotal role of hypoxia inducible factors, HIF-1α and HIF-2α in glioma radioresistance.

### Tumor microenvironment

The brain microenvironment contributes to the radiation responses of gliomas [86]. Jamal et al. [87] built a brain tumor xenograft model initiated from CD133+ GSCs and discovered that γH2AX foci induction after irradiation *in vivo* was significantly reduced in CD133+ glioma cells, compared with CD133- cells in the same tumor. However, there was no difference in γH2AX foci induction or dispersal between CD133+ and CD133- cells after irradiation *in vitro*. Similarly, another study showed that glioma cells irradiated intracerebrally had a greater DDR capacity than the same cells irradiated *in vitro* [88]. These discoveries firmly attested to the influence of the brain microenvironment on gliomas’ radioresistance, especially on GSCs.

Paracrine signals released by endothelial cells can promote proliferation and renewal of GSCs [89]. GSCs co-cultured with tumor microvascular endothelial cells isolated from the same tumor specimen recovered more quickly from radiotherapy [90], suggesting that endothelial paracrine interactions can also contribute to the radioresistance of glioma cells.

The impact of the tumor microenvironment on glioma radioresistance appears to be complex, and there is still limited information available in this regard. Moreover, since the mechanisms behind the increased DDR observed in GSCs in vivo are uncertain, investigating the role of tumor angiogenesis and paracrine signaling might shed much needed light.

### Others factors

Numerous factors, in addition to the ones presented above, have shown to influence the radioresistance of gliomas through modulation of DDR, apoptosis, and cell cycle progression. High expression of neuron-glia antigen 2 (NG2), a glial progenitor marker, was reported in high-grade, compared with low-grade, gliomas [91]. Analysis of patient data and experimental evidence suggest that NG2 may promote radiation resistance by increasing the cell’s antioxidant capacity and the DDR response via upregulation of the oxygen scavenging enzyme PRDX-1 and increased AKT signaling, respectively. Based on expression analyses indicating its correlation with poorer survival, NG2 quantification in glioma may be of important prognostic value [92]. In addition, it was proposed that the interaction between NG2 and OMI/HtrA2, a mitochondrial serine released from damaged mitochondria in response to stress, also contributes to the elevated chemo- and radioresistance of gliomas [93].

Several other genes and proteins, including TRIB1 [94], TPM1 [95], P2X7R [96], EPOR [97], RAD18 [98], and cofilin-1 [99], have been implicated in glioma radioresistance. Further characterization of the molecular mechanisms mediating their actions will reveal whether these proteins could be optimal targets to increase sensitivity during glioma radiotherapy.

## CONCLUSIONS

Radiotherapy is part of the standard treatment for both low-grade and high-grade gliomas. However, as intrinsic or acquired radioresistance underlies the poor radiotherapeutic responses observed especially in glioblastoma patients, overcoming radioresistance is a pressing clinical challenge. As summarized in this review, numerous signaling pathways, proteins and microRNAs that are active on differentiated glioma cells or CSCs have shown to influence radiation resistance.. However, since most of the studies addressing this problem have largely focused on individual pathways or molecules, further research is needed to bring to light the complex signaling network that controls glioma cells’ intrinsic radioresistance. Ideally, combining radiation therapy with radiosensitizing therapies targeting GSCs, or specific microRNAs or signaling pathways selected with basis on each tumor’s molecular profile, would be a more effective approach to reduce primary tumor burden, prevent recurrence, and prolong the survival of glioma patients.
